# Evaluation of visual inspection with acetic acid and Pap smear tests in early cervical cancer screening among women in Meru County, Kenya

**DOI:** 10.4102/ajlm.v15i1.3054

**Published:** 2026-08-05

**Authors:** Mary N. Chege, Stanley Kang’ethe, Scolastica N. Kimani

**Affiliations:** 1 Department of Medical Laboratory Sciences, Medical School, Mount Kenya University, Thika, Kenya; 2 Department of Pathology, Faculty of Medicine, Meru Teaching and Referral Hospital, Meru, Kenya

**Keywords:** cervical cancer screening, visual inspection with acetic acid, Pap smear, colposcopy, women, Kenya

## Abstract

**Background:**

Early cervical dysplasia screening can save women from death resulting from cervical cancer.

**Objective:**

To evaluate and compare the diagnostic performance of the Papanicolaou (Pap) smear and visual inspection with acetic acid (VIA) against histopathology as the reference test.

**Methods:**

We recruited 160 women aged 25–60 years who were undergoing early cervical screenings at the Meru teaching and referral hospital, from June to October 2024. The sensitivity, specificity, positive predictive value (PPV), and negative predictive value (NPV) were calculated by comparing VIA and Pap smear outcomes against histology (gold standard).

**Results:**

Mean age of the study sample was 44.96 years (s.d. = 9.49). Eighteen (11%) had inflammation, 3 (1.9%) had squamous cell carcinoma against 11 (6.9%) detected by histology, and 14 (8.8%) had precancerous lesions. There was no statistical significance between age or number of pregnancies and histology-confirmed cervical lesions. Pap smear had a sensitivity of 100% (95% confidence interval [CI]: 86–100), specificity of 94% (95%CI: 89–97), PPV of 76% (95%CI: 58–89), and NPV of 100% (95%CI: 97–100). In comparison, VIA showed diagnostic performance with sensitivity of 72% (95%CI: 51–88), specificity of 81% (95%CI: 74–88), PPV of 42% (95%CI: 27–58), and NPV of 94% (95%CI: 88–98).

**Conclusion:**

Pap smear showed higher overall diagnostic accuracy than VIA in diagnosing cervical lesions. While less specific, VIA showed a high NPV, suggesting its potential in ruling out disease in low-resource settings. Although age and pregnancy showed no statistical significance with cervical dysplasia, more studies including characteristics are required.

**What this study adds:**

Results provide updated evidence on the relative diagnostic performance of VIA and Pap smear, to guide cervical cancer screening strategies in settings with limited resources.

## Introduction

Cervical cancer and cervical neoplasia are mostly caused by human papillomavirus (HPV) infections, especially those involving high-risk strains of the virus. Various studies have shown that a vast majority of cervical cancer cases worldwide are caused by HPV, a sexually transmitted virus.^1,2^ The danger is much higher for women who are living with HIV, whose risk of cervical cancer is six times greater.^3^ The majority of the 197 distinct subtypes of HPV do not cause any harm, but a tiny percentage do. Variegates 16 and 18 account for approximately 70% of cervical cancer cases.^4^ There are other types that present lower incidences. It usually takes around 10 years after infection with a high-risk oncogenic strain for invasive cervical cancer to occur.

Worldwide, an estimated 38.8% of women are carrying the cervical HPV virus.^1^Among female cancer patients, cervical cancer ranks fourth, and 3.3% of cancer deaths are attributable to it.^5,6^ The worst hit region is Eastern Africa. Here, mortality rate per year in 100 000 women is 34.5 while age-standardised incidence is 25.3. When it comes to cancers affecting women, it comes second worldwide and is still one of the top killers in underdeveloped countries.^7^ Kenya is a low- and middle-income nation, making cervical cancer a big health issue there. Globally, 10.32 million women aged 15 and above could be impacted by the disease. It is still a major risk to reproductive health, especially in areas where people do not have easy access to adequate screening methods.^8^

When it comes to female-specific diseases, cervical cancer ranks high among both the frequency and mortality rates worldwide. This malignancy is a big public health problem, especially in low- and middle-income countries, where it ranks fourth globally. Recent studies show that these resource-limited countries account for around 90% of the expected 342 000 cervical cancer-related fatalities in 2020; with above 604 000 additional cases anticipated.^9^ Furthermore, it ranks as the world’s second most frequent malignancy.^2^ With greater infection rates, developing countries bear a hefty share of the world’s 266 000 yearly victims.^10^As far as female cancers are concerned, sub-Saharan Africa predominates. The high HIV prevalence in the region greatly increases the risk of cervical cancer for women.^11,12^

Although it accounts for just 12% of all cancer occurrences, cervical cancer is the top killer in Kenya.^9^ Pre-cancers can fortunately be treated and screened effectively to prevent cervical cancer.^13^ Enhancing capacity, harmonising, and optimising cancer screening, diagnosis, detection, and treatment are imperative. Inadequacy of health literacy, poverty, problems with infrastructure, among many potential hurdles often hinder the perception of screening’s importance and challenging health objectives towards screening for cervical cancer.^13,14^ The only certain approach to manage and prevent cervical cancer is to detect and treat early. Meru Teaching and Referral Hospital (MeTRH), like many healthcare facilities in Kenya, operates in a resource-limited setting. Although Papanicolaou (Pap) smear and visual inspection with acetic acid (VIA) screening methods are available in this facility, there is limited comparative data on their effectiveness in detecting early cervical dysplasia in this population. The study aimed to address the following primary and secondary objectives.

## Study objectives and hypotheses

### Primary objective

To evaluate and compare the diagnostic performance of VIA and the Pap smear, using histopathology (haematoxylin and eosin staining) as the reference standard, for detecting cervical precancer and cancer among women aged 25–60 years in Meru County, Kenya.

### Secondary objectives

- To estimate and compare the diagnostic accuracy measures (sensitivity, specificity, positive predictive value [PPV], negative predictive value [NPV], and overall accuracy) for VIA and Pap smear.
- To determine the level of agreement between each screening test and histopathology using Cohen’s kappa statistic.
- To assess the influence of demographic and reproductive factors (such as age, gravida, and parity) on histology-confirmed cervical lesions using logistic regression analysis.

### Study hypothesis

We hypothesised that the Pap smear would demonstrate higher diagnostic accuracy than VIA in identifying histology-confirmed cervical precancer and cancer, and that older age and higher parity may be associated with increased likelihood of cervical lesions.

## Methods

### Ethical considerations

The study ethical approval was sought from the ethical review committee of Mount Kenya University (reference number MKU/ISERC/3548). The research permit was obtained from National Commission for Science, Technology, and Innovation under license number NACOSTI/P/24/34592. Permission to access clients for data collection was approved by the Meru Director of Medical Services. Prior to their participation, all subjects were explained the purpose of the study and were required to give a written consent. Participation in this study was completely voluntary. As a precaution, data privacy was maintained by using password-protected devices only accessible to the principal investigator.

### Study area

The research study was done at MeTRH in Meru County from June to October 2024. This is a level 6 facility that also serves nearby counties including Isiolo, Laikipia, Marsabit, Tharaka Nithi, and Embu. Meru County is one of the 47 Counties in Kenya with a population of about 1.55 million, 777 975 of whom are female.^15^ Meru Town, where MeTRH is situated, lies on the north-eastern slopes of Mount Kenya, at 37.649803° east and 0.047035° north.

### Study design and sampling method

The study used a cross-sectional design aimed at comparing data on cervical cancer screening methods. A consecutive sampling technique was employed to select 160 participants who met the selection criteria. All women who visited MeTRH for early cervical screening during the study period and met the inclusion criteria were invited to participate. Women were included consecutively as they presented until the required sample size was reached, ensuring that every eligible attendee had an equal opportunity to participate. The sample size was calculated using the formula for an infinite population.^16^

#### Sample size determination

In this study we were unsure of the number of women visiting MeTRH for early cervical screening; we assumed the population is infinite. Therefore, the sample size (*n*) was calculated using Cochran’s formula (see Equation 1) for an infinite population^16^:


$$n=\frac{Z^{2}\cdot p\cdot (1-p)}{e^{2}}$$ [Eqn 1]

Where:

*Z* = *Z*-value (e.g., 1.96 for a 95% confidence level)

*p* = Estimated proportion of the population (0.5 is used for maximum variability)

*e* = Margin of error (e.g. 0.0775 for ± 7.75%)

We chose a margin of error of 7.75% (*e* = 0.0775) because of resource constraints in this study.^17^

Plugging these values into the formula in Equation 2:


$$\begin{array}{c} n=\frac{(1.96{)}^{2}\cdot 0.5\cdot (1-0.5)}{(0.0775{)}^{2}}\\ n=\frac{3.8416\cdot 0.5\cdot 0.5}{0.00600625}\\ n=\frac{0.9604}{0.00600625}\\ n\approx 159.9\\ n\approx 160 \end{array}$$ [Eqn 2]

#### Recoding of test variables

To perform diagnostic accuracy analysis, the results from VIA, Pap smear, and histology were recorded into binary factor variables with positive and negative categories. Histology, the reference standard, was coded as positive for confirmed precancerous or cancerous lesions (squamous cell carcinoma, cervical intraepithelial neoplasia I–III, adenocarcinoma in situ), and those cases where no abnormal Pap smear results were obtained (No evidence of malignancy). Pap smear was coded negative for intraepithelial lesion or malignancy (NILM) and atypical squamous cell of undetermined significance, and positive for atypical squamous cells, cannot exclude high-grade intraepithelial lesions, low-grade squamous intraepithelial lesion, high-grade squamous intraepithelial lesion, and atypical glandular cells, not otherwise specified, allowing conservative classification in sensitivity analysis. Visual inspection with acetic acid was coded negative for ‘Negative’ findings and positive for all other outcomes, including ‘Suspicious’ and ‘Positive’, which is consistent with clinical practice. All recoded variables were converted to factors with levels 0 = negative and 1 = positive for computation of sensitivity, specificity, PPV, NPV, and Kappa against histology.

#### Inclusion and exclusion

This study sought to recruit women between 25–60 years old seeking cervical screening services at MeTRH. The investigator explained the qualities needed, procedures, including potential risks and benefits, and obtained informed consent from qualified participants. However, several factors determined who to exclude from the study. Some of these included women who were already diagnosed with cervical cancer, who had undergone total hysterectomy, those below 25 years or above 60 years of age, and those who had a cervical biopsy or cryotherapy or any other intrusive procedure done in the past 6 weeks.^18,19^ Similarly, those who, at the time of screening, were on menses or had vaginal bleeding were also excluded from the study.

### Data and sample collection procedures

The participants were given instructions to follow before the day of sample collection. No special preparation was needed for a Pap test, but clients were advised to avoid such things as douching, applying tampons, jellies, moisturisers, or using spermicidal foams and vaginal medicines for 7 days or more, and sexual intercourse for 2 days before the test.^20^ Socio-demographic data such as parity, age, and screening pattern, were collected using a semi-structured questionnaire.

First, the participants were advised to empty their bladder before the procedure was done. Then one by one, they were requested to undress from the waist down, then lie in a lithotomy position on a prepared examination couch and place their feet in stirrups while spread. A disposable non-lubricated plastic speculum was gently inserted into the vagina to allow viewing of the cervix.^21^ An Ayer’s spatula was used to gently scrape cells from the ectocervix and endocervical canal.^18,22^ The obtained material was spread on a labelled glass slide and was immediately fixed in a coupling jar containing 95% absolute alcohol. The prepared fixed smears were sent to the pathology laboratory in MeTRH for staining using Pap stain and microscopic examination.^23,24^ The participants were advised to come back for the results after 2 weeks, at a specified date. Samples for histological examination were obtained through colposcopy from those found with abnormal results.

For colposcopy, the participants were directed to lie on an examination table and a Colposcope was used to provide illumination and magnification to allow targeted examination of the cervix and the vaginal walls. A solution of 3% acetic acid was applied over the cervix and visualised by naked eye after 1 min for patches of aceto-white, which indicated abnormal cells.^21^ Alternatively, Lugol’s solution (iodine) was used to further differentiate normal and abnormal cells whereby areas with normal cells stained brown, while abnormal areas appeared yellow. If positive and suspicious areas were identified, a small tissue sample (biopsy) was taken for processing and analysis in the histology department in MeTRH. Quality control was maintained throughout by running known positive and negative specimen samples together with the test samples.

The procedures were performed guided by the standard operating procedures, trained personnel, and the Bethesda system for reporting. Examinations and reporting were done by a cytotechnologist. A second cytotechnologist re-examined them blindly to maintain reliability. The results of the two were compared and those identified as abnormal were reassessed by a pathologist to validate the diagnosis. Depending on biopsy outcome, the participants were referred to a consultant in oncology department for counselling, follow-up, and targeted treatment options like loop electrosurgical excision procedure to ensure proper healing.

### Data analysis

Study data were analysed using computer software R version 4.3. Qualitative data were presented as frequencies and percentages. Quantitative data were presented as mean and standard deviation. A 2 × 2 contingency table was used to calculate sensitivity, specificity, PPV, NPV and diagnostic accuracy of VIA and Pap smear in diagnosing cervical cancer, against histopathology as gold standard.^25^ Logistic regression analysis was performed to obtain the odds ratios, corresponding 95% confidence interval (CI), and *p* values.

## Results

### Demographic characteristics of the study population

The study included 160 participants, aged between 25–60 years. The largest proportion of these women were between the age of 35–44 years (*n* = 55, 34.4%) followed by those between 45–54 years (*n* = 49; 31%) and the least were those aged 60+ years (*n* = 13; 8%). This indicated that the sample represented women in their reproductive and perimenopausal years. The cohort was predominantly multi-gravid, with about 99.4% (*n* = 159) had experienced at least one pregnancy. Notably, over two-third of the participants (68%) reported more than two pregnancies, among which about 61% were living children, suggesting a population with high parity. However, approximately 24% (*n* = 39) of the participants experienced at least one pregnancy loss (Table 1).

**TABLE 1:** Demographic characteristics of the study population for women at Meru teaching and referral hospital from June to October 2024.

Demographic characteristics	*n*	%
**Age group (years)**		
25–34	24	15.0
35–44	55	34.4
45–54	49	30.6
55–59	19	11.9
60+	13	8.1
**Gravidity (number of pregnancies)**		
0	1	0.6
1–2	50	31.3
3+	109	68.1
**Number of living children**		
0	1	0.6
1–2	61	38.1
3+	98	61.3
**Number of pregnancy losses**		
0	121	75.6
1	33	20.6
2+	6	3.8

### Diagnostic evaluation tests

A majority of participants were negative (NILM) with Pap smear, accounting for 68.1% (*n* = 109). Eighteen (11%) of the participants were diagnosed with inflammatory variations associated with atypical squamous cell of undetermined significance. Squamous cell carcinoma was identified in only 3 (1.9%) cases by Pap smear. Twenty-six precancerous lesions cases were detected: High-grade squamous intraepithelial lesion, 9 (5.6 %); high-grade intraepithelial lesions, 8 (5%); low-grade squamous intraepithelial lesion, 9 (5.6%). Four (2.5%) participants tested positive for atypical glandular cells, not otherwise specified. Histology results showed that 126 (78.8%) participants were NILM. Squamous cell carcinoma was detected in 11 (6.9%) cases by histology. Cervical intraepithelial neoplasia I, II, III and adenocarcinoma in situ were detected in 14 (8.8%) participants, as shown in Table 2.

**TABLE 2:** Cases distribution based on Pap smear, visual inspection with acetic acid and histopathological findings for women at Meru teaching and referral hospital from June to October 2024.

Distribution of conditions	*n*	%
**Pap (160)**		
Negative		
NILM	109	68.1
ASC-US	18	11.3
Negative		
HSIL	9	5.6
LSIL	9	5.6
ASC-H	8	5.0
AGC-NOS	4	2.5
SCC	3	1.9
**VIA (160)**		
Positive	35	22.0
Negative	117	74.0
Suspicious	8	5.0
**Histopathological examination**		
Normal	126	78.8
CIN I	3	1.9
CIN II	5	3.1
CIN III	4	2.5
AIS	2	1.3
SCC	11	6.9

Pap, Papanicolaou; VIA, Visual inspection with acetic acid; NILM, negative for intraepithelial lesion or malignancy; ASC-US, atypical squamous cell of undetermined significance; HSIL, high-grade squamous intraepithelial lesion; LSIL, low-grade squamous intraepithelial lesion; ASC-H, atypical squamous cells, cannot exclude high-grade intraepithelial lesions; AGC-NOS, atypical glandular cells, not otherwise specified; CIN, cervical intraepithelial neoplasia; AIS, adenocarcinoma in situ; SCC, squamous cell carcinoma.

### Participant flow and verification

All eligible participants were screened using VIA and Pap smear screening methods. Histology and biopsy were performed conditionally, where only those participants with abnormal Pap smear results were assessed, in accordance with standard clinical management pathways. Histology was not performed on participants with NILM or atypical squamous cell of undetermined significance results, but they were treated as negative for histology in the sensitivity analysis.

By using this kind of classification, VIA showed moderate sensitivity and specificity relative to the histology reference. It had a high NPV, suggesting good capability to rule out the disease. On the other hand, Pap smear demonstrated higher diagnostic performance and stronger agreement with histology.

### Diagnostic evaluation

The diagnostic performance of VIA and Pap smear was evaluated against histology as the gold standard (Table 3 and Table 4).

**TABLE 3:** Visual inspection vs histology for women at Meru teaching and referral hospital from June to October 2024.

VIA result	Histology negative (0)	Histology positive (1)	Total
VIA Negative (0)	110 (TN)	7 (FN)	117
VIA Positive (1)	25 (FP)	18 (TP)	43
Total	135	25	160

VIA, visual inspection with acetic acid; TN, true negative; FN, false negative; FP, false positive; TP, true positive.

**TABLE 4:** Pap smear vs histology for women at Meru teaching and referral hospital from June to October 2024.

Pap smear result	Histology negative (0)	Histology positive (1)	Total
Pap negative (0)	127 (TN)	0 (FN)	127
Pap positive (1)	8 (FP)	25 (TP)	33
Total	135	25	160

Pap, Papanicolaou; TN, true negative; FN, false negative; FP, false positive; TP, true positive.

Overall, VIA demonstrated moderate diagnostic accuracy, with a sensitivity of 72% (95%CI: 51–88) and a specificity of 67% (95%CI: 30–93). The test yielded a relatively high PPV of 86% (95%CI: 64–97), but a low NPV of 46% (95%CI: 19–75). Agreement between VIA and histology was moderate, with a Cohen’s kappa of 0.34.

In comparison, the Pap smear showed a perfect sensitivity of 100% (95%CI: 86–100), indicating that it correctly identified all histology-positive cases in this sample. However, its specificity was low at 11% (95%CI: 0–48), suggesting a high number of false-positive results. The Pap smear had a PPV of 76% (95%CI: 58–89) and a perfect NPV of 100% (95%CI: 3–100), which was substantially higher than that observed for VIA. Despite its high sensitivity, agreement between Pap smear and histology was only slight, with a Cohen’s kappa of 0.16 (see Table 5).

**TABLE 5:** Visual inspection and Pap smear results for women at Meru teaching and referral hospital from June to October 2024.

Test	Sensitivity	95%CI	Specificity	95%CI	PPV	95%CI	NPV	95%CI	Cohen’s kappa
VIA	0.72	0.51–0.88	0.81	0.74–0.88	0.42	0.27–0.58	0.94	0.88–0.98	0.41
Pap smear	1.00	0.86–1.00	0.94	0.89–0.97	0.76	0.58–0.89	1.00	0.97–1.00	0.83

CI, confidence interval; VIA, visual inspection with acetic acid; Pap, Papanicolaou; PPV, positive predictive value; NPV, negative predictive value.

### Impact of age and parity on cervical lesions

In the logistic regression analysis, neither age nor parity was significantly associated with histology-confirmed cervical lesions. Increasing age showed a small, non-significant increase in the odds of a positive histology result (*β* = 0.034; odds ratio = 1.04, 95%CI: 0.98–1.09; *p* = 0.208), indicating that each additional year of age was associated with approximately a 3% increase in odds, although this effect was not statistically significant. Similarly, parity was not a significant predictor (*β* = 0.154; odds ratio = 1.17, 95%CI: 0.84–1.61; *p* = 0.347), suggesting that a higher number of pregnancies was associated with slightly increased odds of disease, but with wide confidence intervals crossing 1. Overall, these findings indicate that age and gravida did not independently predict histological outcomes in this study population (see Table 6).

**TABLE 6:** Logistic regression analysis for women at Meru teaching and referral hospital from June to October 2024.

Predictor	Estimate (*β*)	OR	95%CI lower	95%CI upper	*p*
Intercept	−3.804	0.022	0.0019	0.20	0.0012
Age	0.034	1.035	0.982	1.093	0.2084
Parity	0.154	1.166	0.841	1.606	0.3466

OR, odds ratio; CI, confidence interval.

Although age and parity were not statistically significant predictors in this analysis, further studies with larger sample sizes are recommended to better explore and clarify their potential association with histology-confirmed cervical lesions.

## Discussion

The study also discovered a non-significant association between higher parity and an increased likelihood of cervical dysplasia. The likelihood of cervical dysplasia being positive increased for women who gave birth three times or more. Nevertheless, beyond seven childbirths, the distribution of abnormalities did not remarkably increase, indicating that factors beyond parity alone may contribute to cervical changes. Diagnostic evaluation revealed that Pap smear offered better sensitivity, specificity, PPV, and NPV, making it the most effective test for confirming cervical dysplasia and ruling out false negatives compared to VIA. However, in high-prevalence settings, Pap smear may still pose a risk of false negatives, demanding follow-up testing for patients with negative results to ensure accurate diagnosis. The research found that the frequency of cervical anomalies increased with age, particularly in the lower age group. This further demonstrates why age-specific programmes and early screening are so beneficial. Improved surveillance and early interventions for women 40 years and above are crucial to facilitating early diagnosis and treatment of cervical dysplasia before it progresses to cervical cancer. Notably, cervical dysplasia was also diagnosed across all age groups, emphasising the vital role of early continuous screening even in women of young age. Additionally, the confirmed squamous cell carcinoma cases support the necessity of early screening, diagnosis, and intervention among all women above 24 years of age.

### Diagnostic performance of visual inspection with acetic acid and Papanicolaou smears

In comparison to VIA, Pap smears were more sensitive and specific, according to the results of this investigation, making it a more reliable early screening tool. These results differ from previous studies that discovered higher sensitivity for VIA but lower specificity.^25,26,27^ Pap smear, as shown by our findings, is an important tool for routinely screening cervical cancer. Additionally, when Pap smear and HPV tests are not an option, VIA should be considered as an additional, viable screening tool where resources are limited.^28^ However, a meta-analysis found that VIA sensitivity ranged between 65% and to 95%, while Pap smear sensitivity was found to be lower, ranging between 50% and to 75%.^29^ Nevertheless, in this study, Pap smear (93%) outperformed VIA (83%) in detecting cervical dysplasia, aligning with a previous study, and underscored the importance of cytotechnician proficiency in enhancing Pap smear accuracy.^30^

In this study, possible explanations for the superiority of Pap smear in both sensitivity and specificity could be due to the small sample size, low disease prevalence, or its higher accuracy in detecting high-grade malignancy. Several studies have argued that conducting Pap smear under optimal conditions can surpass VIA in cervical diagnostic performance.^31^ Moreover, researchers have shown that liquid-based cytology is more sensitive than the traditional Pap smear, which may be the reason for the observed differences.^32^ Conversely, VIA’s lower accuracy in this study could be ascribed to inter-observer variability, since interpretation of VIA test is individual and dependent on the medical professional’s training and expertise. A study on acetic acid application variability and differences in lesion identification clarify why VIA performed worse than in some previous studies performed in low-income settings.^31^

### Age and cervical dysplasia

In this study, the findings revealed that the prevalence of cervical dysplasia was detected across all age groups. Some earlier studies demonstrate that the disease had a peak at 35–45 years.^33^ Older women are at higher risk because of persistent HPV infections, delayed screening, or a lack of follow-up care after they reach menopause. In a recent study, women below the age of 30 were found to have the highest prevalence of HPV infection, but persistent infection and progression to dysplasia were found to be more prevalent in middle-aged women.^33^ Nevertheless, our findings concur with a similar one which revealed that postmenopausal women still have a chance of developing high-grade lesions after 50 years,^32^ highlighting the need for ongoing monitoring. This shift in peak prevalence may be a result of cohort effects where aged women could not have taken part in early cervical cancer screening programmes as frequently as needed. In addition, latent high-risk HPV infections, although not evaluated in this study, might have been reactivated by immune responses, influencing late-onset cervical dysplasia.^32,33,34^ The findings emphasise the necessity for screening policies that do not prematurely exclude women going into their 50s. This is because the threat of cervical precancerous lesions is quite substantial for this age group.

### Parity and cervical dysplasia

The current study revealed a non-significant association between parity and cervical dysplasia. This is in line with other findings that reported that hormonal changes, cervical trauma, and frequent, prolonged HPV exposure during childbirth may promote neoplastic transformation.^35^ Our findings also agree with those of other researchers who established in their study of sub-Saharan African women that five or more births had nearly double risk of cervical intraepithelial neoplasia-III compared to nulliparous women.^36^ However, this relationship was weaker in Western populations, perhaps because of differences in contraceptive use, HPV vaccination, and early screening programmes.^37^

### Strength and limitations

A key limitation of this study is the potential for partial verification or work-up bias, which happens since histopathology was limited to participants with abnormal Pap smear findings. Participants with NILM or atypical squamous cell of undetermined significance recorded as negative for histology despite not undergoing biopsy for sensitivity analysis. This may have introduced mis-classification if underlying lesions were present but not verified. This may result in overestimation of diagnostic accuracy, and especially sensitivity and NPV, and requires cautious interpretation. Future studies should consider using universal histological verification or apply an alternative statistical method that can account for verification bias and improve the precision of diagnostic accuracy estimates. Because this was a cross-sectional study, it couldn’t track changes over time. There was potential inter-observer variability in VIA interpretation. The sample was obtained from a single clinical setting which limits its capability to be generalised to a bigger population. Awareness issues, as well as cultural barriers such as stigma and embarrassment surrounding gynaecological examination was also a limitation. The limited representation of younger women in the study may impact age-specific analyses and proposes the necessity for targeted recruitment strategies to achieve broader age diversity from adolescent girls to older women across different settings.

### Conclusion

It is clear from this study’s results that engaging in early cervical screening, using a larger sample size, and maintaining diagnostic accuracy would work well in improving early detection, treatment, and overall cervical cancer prevention. The findings confirm that Pap smear is more sensitive and specific, while VIA has high sensitivity but is less specific. Further studies are needed to explore longitudinal follow-ups and molecular HPV testing to enhance screening accuracy. The findings suggest that a suspicious or positive VIA result should be confirmed using Pap smear or histology to lower the number of false positives. Nevertheless, training and standardisation of VIA procedures need enhancement to improve specificity. The implications of this study are to reinforce the need for policy-driven cervical cancer screening strategies that focus on middle-aged and older women at higher risk, while making sure early diagnosis efforts remain accessible for all age groups starting from adolescent girls.

## References

[1] Kinoti NJ, Muturi M, Kamau L, Lwembe R. Human Papilloma virus types prevalence and their association with cervical dysplasia among HIV and non-HIV infected women attending reproductive health clinics in Eastern Kenya. Afri Health Sci. 2022;22(1):106–114. 10.4314/ahs.v22i1.14 PMC9382485 36032490

[2] Cohen CM, Wentzensen N, Castle PE, et al. Racial and ethnic disparities in cervical cancer incidence, survival, and mortality by histologic subtype. J Clin Oncol. 2023;41(5):1059–1068. 10.1200/JCO.22.01424 36455190 PMC9928618

[3] Stelze D, Tanaka LF, Lee KK, et al. Estimates of the global burden of cervical cancer associated with HIV. Lancet. 2020;9(2):E161–E169. 10.1016/S2214-109X(20)30459-9 PMC7815633 33212031

[4] Swase TD, Fasogbon IV, Eseoghene IJ, et al. The impact of HPV/HIV co-infection on immunosuppression, HPV genotype, and cervical cancer biomarkers. BMC Cancer. 2025;25:202. 10.1186/s12885-025-13516-2 39910495 PMC11796042

[5] Canfell K, Kim JJ, Brisson M, et al. Mortality impact of achieving WHO cervical cancer elimination targets: A comparative modelling analysis in 78 low-income and lower-middle-income countries. Lancet. 2020;395(10224):591–603. 10.1016/S0140-6736(20)30157-4 32007142 PMC7043006

[6] Ferlay J, Ervik M, Lam F, et al. Global Cancer Observatory (GLOBOCAN 2022): Cancer today (version 1.1) [homepage on the Internet]. Lyon: International Agency for Research on Cancer; 2024 [cited 2025 Mar 28]. Available from: https://gco.iarc.fr/today

[7] Aziz H, Sattar AA, Mahmood H, Fatima S, Khurshid M, Faheem M. Prevalence of HPV types in HIV-positive and negative females with normal cervical cytology or dysplasia. J Clin Lab Anal. 2023;37(4):1–7. 10.1002/jcla.24851 PMC10020845 36807631

[8] Brisson M, Kim JJ, Canfell K, et al. Impact of HPV vaccination and cervical screening on cervical cancer elimination: A comparative modelling analysis in 78 low-income and lower-middle-income countries. Lancet. 2020;395(10224):575–590. 10.1016/S0140-6736(20)30068-4 32007141 PMC7043009

[9] Sung H, Ferlay J, Siegel RL, et al. Global cancer statistics 2020: GLOBOCAN estimates of incidence and mortality worldwide for 36 cancers in 185 countries. CA Cancer J Clin. 2021;71(3):209–249. 10.3322/caac.21660 33538338

[10] Modibbo F, Iregbu KC, Okuma J, et al. Randomized trial evaluating self-sampling for HPV DNA based tests for cervical cancer screening in Nigeria. Infect Agent Cancer. 2017;12:11. 10.1186/s13027-017-0123-z 28184239 PMC5294803

[11] UNAIDS. The GAP report. Geneva: Joint United National Program on HIV/AIDS (UNAIDS); 2014.

[12] Denny LA, Franceschi S, De Sanjose S, Heard I, Moscicki AB, Palefsky J. Human Papillomavirus, human immunodeficiency virus and immunosuppression. Vaccine. 2012;30(suppl 5):F168–F174. 10.1016/j.vaccine.2012.06.045 23199960

[13] Sudenga SL, Rositch AF, Otieno A, Smith JS. Knowledge, attitudes, practices, and perceived risk of cervical cancer among Kenyan women: Brief report. Int J Gynecol Cancer. 2013;23(5):895–899. 10.1097/IGC.0b013e31828e425c 23694983 PMC3662490

[14] Denny L, De Sanjose S, Mutebi M, et al. Interventions to close the divide for women with breast and cervical cancer between low-income and middle-income countries and high-income countries. Lancet. 2016;389(10071):861–870. 10.1016/S0140-6736(16)31795-0 27814963

[15] Koliopoulos G, Nyaga VN, Santesso N, et al. Cytology versus HPV testing for cervical cancer screening in the general population. Cochrane Database Syst Rev. 2017;8(8):CD008587. 10.1002/14651858.CD008587.pub2 28796882 PMC6483676

[16] Cochran WG. Sampling techniques (3rd ed.). New York, NY: John Wiley & Sons; 1977.

[17] Bartlett JE, Kotrlik JW, Higgins CC. Organizational research: Determining appropriate sample size in survey research. Inf Technol Learn Perform J. 2001;19:43–50.

[18] Kamal M. Pap Smear collection and preparation: Key points. Cytojournal. 2022;19:24. 10.25259/CMAS_03_05_2021 35510105 PMC9063692

[19] Mungo C, Osongo CO, Ambaka J, Omoto J, Cohen CR. Efficacy of thermal ablation for treatment of biopsy-confirmed high-grade cervical precancer among women living with HIV in Kenya. Int J Cancer. 2023;153(12):1971–1977. 10.1002/ijc.34737 37715464 PMC11081005

[20] Jhungran A, Russell AH, Seiden MV, et al. Chapter 84: Cancers of the cervix, vulva, and vagina. In: Niederhuber JE, Armitage JO, Doroshow JH, Kastan MB, Tepper JE, editors. Abeloff’s clinical oncology. 6th ed. Philadelphia, PA: Elsevier; 2020.

[21] Koss LG, Melamed MR, editors. The normal female genital tract. In: Koss’ diagnostic cytology and its histopathologic bases. 5th ed. Philadelphia, PA: Lippincott Williams and Wilkins; 2006, pp. 183–225.

[22] Koss LG, Melamed MR, editors. Laboratory techniques. In: Koss’ diagnostic cytology and its histopathologic bases. 5th ed. Philadelphia, PA: Lippincott Williams and Wilkins; 2006, pp. 1569–1634.

[23] Belgaumi UI, Shetty P. Leishman giemsa cocktail as a new, potentially useful cytological technique comparable to Papanicolaou staining for oral cancer diagnosis. J Cytol. 2013;30:18–22. 10.4103/0970-9371.107507 23661935 PMC3643356

[24] Goel G, Halder A, Joshi D, Anil AC, Kapoor N. Rapid, Economic, Acetic Acid Papanicolaou Stain (REAP): An economical, rapid, and appropriate substitute to conventional Pap stain for staining cervical smears. J Cytol. 2020;37(4):170–173. 10.4103/JOC.JOC_89_20 33776256 PMC7984519

[25] Adnan Z, Khan AA, Nayyar S, Nayyar A, Anwar Z, Ahsan S. Diagnostic accuracy of Visual Inspection with Acetic Acid (VIA) and PAP smear in diagnosing cervical cancer, taking histopathology as gold standard. J Soc Obstet Gynaecol Pak. 2021;11(1):36–40.

[26] Denny L, Kuhn L, De Souza M, et al. Screen-and-treat approaches for cervical cancer prevention in low resource settings. JAMA. 2005;294(17):2173–2181. 10.1001/jama.294.17.2173 16264158

[27] Mremi A, Mchome B, Mlay J, et al. Performance of HPV testing, Pap smear and VIA in women attending cervical cancer screening in Kilimanjaro region, Northern Tanzania: a cross-sectional study nested in a cohort. BMJ Open. 2022;12(10):e064321. 10.1136/bmjopen-2022-064321 PMC9628663 36316070

[28] Sarbhai V. & Aggarwal S. Cervical cancer screening: Comparing PAPs smear with VIA/VILI in semiurban women of Delhi. Ind J Med Paediatr Oncol 2025; 46:200–206. 10.1055/s-0044-1800949

[29] Lohiya A, Daniel RA, Kumar D, et al. Effectiveness of Visual Inspection with Acetic Acid (VIA) screening on cervical cancer mortality and incidence – A systematic review and meta-analysis. Asian Pac J Cancer Prev. 2022;23(2):399–407. 10.31557/APJCP.2022.23.2.399 35225450 PMC9272618

[30] Nayar R, Wilbur DC. The Pap test and Bethesda 2014. Cancer Cytopathol. 2015;123(5):271–281. 10.1016/j.jasc.2015.01.002 25931431

[31] Bhattacharyya AK, Nath JD, Deka H. Comparative study between pap smear and visual inspection with acetic acid (via) in screening of CIN and early cervical cancer. J Midlife Health. 2015;6(2):53–58. 10.4103/0976-7800.158942 26167054 PMC4481740

[32] Arbyn M, Simon M, Peeters E, et al. 2020 list of human papillomavirus assays suitable for primary cervical cancer screening. Clin Microbiol Infect. 2021;27:1083–1095. 10.1016/j.cmi.2021.04.031 33975008

[33] Rayner M, Welp A, Stoler MH, Cantrell LA. Cervical cancer screening recommendations: Now and for the future. Healthcare (Basel). 2023;11(16):2273. 10.3390/healthcare11162273 37628471 PMC10454304

[34] Castellsagué X, Muñoz N, Pitisuttithum, P. et al. End-of-study safety, immunogenicity, and efficacy of quadrivalent HPV (types 6, 11, 16, 18) recombinant vaccine in adult women 24–45 years of age. Br J Cancer. 2011;105(1): 28–37. 10.1038/bjc.2011.185 21629249 PMC3137403

[35] Muñoz N, Franceschi S, Bosetti C, et al. The role of parity and HPV in cervical cancer risk: A pooled analysis of case-control studies. Lancet Oncol. 2002;359:1093–1101. 10.1016/S0140-6736(02)08151-5 11943256

[36] Bray F, Laversanne M, Sung H, Ferlay J, Siegel RL, Soerjomataram I, Jemal A. Global cancer statistics 2022: GLOBOCAN estimates of incidence and mortality worldwide for 36 cancers in 185 countries. CA Cancer J Clin. 2024;74(3):229–263. 10.3322/caac.21834 38572751

[37] Ferlay J, Ervik M, Lam F, et al. Global cancer observatory: Cancer today [homepage on the Internet]. Lyon: International Agency for Research on Cancer; 2024 [cited 2025 Jul 23]. Available from: https://gco.iarc.who.int/today

